# Characterisation of exogenous folate transport in *Plasmodium falciparum*

**DOI:** 10.1016/j.molbiopara.2007.04.002

**Published:** 2007-07

**Authors:** Ping Wang, Qi Wang, Paul F.G. Sims, John E. Hyde

**Affiliations:** Manchester Interdisciplinary Biocentre, Faculty of Life Sciences, University of Manchester, 131 Princess Street, Manchester M1 7DN, United Kingdom

**Keywords:** BCECF-AM, 2′,7′-bis-(2-carboxyethyl)-5,6-carboxylfluorescein acetoxymethyl ester, DHFR, dihydrofolate reductase, DHPS, dihydropteroate synthase, DNP, 2,4-dinitrophenol, pABA, *p*-aminobenzoic acid, MTX, methotrexate, NPP, new permeability pathway(s), PBS, phosphate-buffered saline, PPPK, 6-hydroxymethylpterin pyrophosphokinase, PYR, pyrimethamine, SDX, sulfadoxine, THF, tetrahydrofolate, TS, thymidylate synthase, Folate metabolism, Folate salvage, Malaria parasites, Metabolic inhibitors, Proton symport, Transporters

## Abstract

Folate salvage by *Plasmodium falciparum* is an important source of key cofactors, but little is known about the underlying mechanism. Using synchronised parasite cultures, we observed that uptake of this dianionic species against the negative-inward electrochemical gradient is highly dependent upon cell-cycle stage, temperature and pH, but not on mono- or divalent metal ions. Energy dependence was tested with different sugars; glucose was necessary for folate import, although fructose was also able to function in this role, unlike sugars that cannot be processed through the glycolytic pathway. Import into both infected erythrocytes and free parasites was strongly inhibited by the anion-channel blockers probenecid and furosemide, which are likely to be acting predominantly on specific folate transporters in both cases. Import was not affected by high concentrations of the antifolate drugs pyrimethamine and sulfadoxine, but was inhibited by the close folate analogue methotrexate. The pH optimum for folate uptake into infected erythrocytes was 6.5–7.0. Dinitrophenol and nigericin, which strongly facilitate the equilibration of H^+^ ions across biological membranes and thus abolish or substantially reduce the proton gradient, inhibited folate uptake profoundly. The ATPase inhibitor concanamycin A also greatly reduced folate uptake, further demonstrating a link to ATP-powered proton transport. These data strongly suggest that the principal folate uptake pathway in *P. falciparum* is specific, highly regulated, dependent upon the proton gradient across the parasite plasma membrane, and is likely to be mediated by one or more proton symporters.

## 1 Introduction

Reduced folates are essential cofactors for one-carbon transfer reactions, including the conversion of dUMP to dTMP, which is a prerequisite for DNA synthesis. Because of this, the folate pathway has long been a target for drugs deployed against rapidly reproducing cells such as cancers and a range of microbial pathogens. Whereas most microorganisms can synthesise the folates they need from the simple precursors GTP, *p*-aminobenzoic acid (pABA) and glutamate, higher animals (but not plants) have lost the ability to do this and depend on dietary intake of pre-formed folate as an essential nutrient. The human malaria parasite *Plasmodium falciparum* is able to exploit both of these routes [1–4]. Thus, it can utilise folate provided in culture medium in vitro or salvaged from the host plasma in vivo on the one hand, or convert the above precursors de novo into folate derivatives on the other, a characteristic also shared by the related apicomplexan parasite *Toxoplasma gondii* [5,6]. The relative importance of the biosynthetic and salvage pathways across the complete life cycle in vivo and the interplay between them is poorly understood, although existing data support the view that both are necessary for healthy propagation of the parasite, at least in the erythrocytic stages [7].

An important aspect of exogenous folate utilisation is the machinery and mechanism(s) by which folate is imported into the parasite. The highly polar nature of folate derivatives suggests that salvage must employ some kind of mediated transport process, as diffusion alone across the membrane is likely to be far too inefficient. Moreover, folate molecules are dianionic at physiological pH and must be imported into the parasite against an inwardly negative electropotential that has been measured as ca. −95 mV in *P. falciparum* [8]. Although the transport of other key molecules, such as pantothenate, lactate, glucose and choline, has been investigated [9–13], there has been no detailed study to date of this aspect of folate metabolism in *P. falciparum*, a better understanding of which might lead to new ways of inhibiting parasite growth. A preliminary approach to this end has been taken by demonstrating that the anti-gout drug, probenecid, which among other things inhibits folate transport in mammalian cells [14], can increase the sensitivity of *P. falciparum* to antifolate inhibitors [15]. Here, we demonstrate that folate uptake by this parasite is a regulated process that is critically dependent upon provision of glucose or another sugar that can proceed through the glycolytic pathway, that the existence of a pH gradient across the plasma membrane is also required for efficient transport, and that folate is transported principally by a proton-symport mechanism.

## 2 Materials and methods

### 2.1 Chemicals

[3′,5′,7,9-^3^H]folic acid, 24 Ci mmol^−1^, 1 mCi ml^−1^ was from Amersham, UK, [3′,5′,7,9-^3^H]folinic acid (26 Ci mmol^−1^, 1 mCi ml^−1^) and [3′,5′,7,9-^3^H]5-methyltetrahydrofolic acid (44 Ci mmol^−1^, 1 mCi ml^−1^) were both from Moravek, California. Folic acid, folinic acid, 5-methyltetrahydrofolic acid, 2,4-dinitrophenol, concanamycin A, d- and l-glucose, d-fructose, d-xylose, d-galactose, 6-deoxy-d-glucose, probenecid and furosemide were all purchased from Sigma, UK. Note that we use the term ‘folate’ generically to indicate derivatives of the folate family of molecules regardless of their oxidation state, modifications at the 5 and 10 positions or polyglutamation status.

### 2.2 Parasite culture

*P. falciparum* was routinely cultured under 1% O_2_, 3% CO_2_, 96% N_2_ in RPMI 1640 medium, supplemented with d-glucose (22 mM final concentration), hypoxanthine (36 mM), HEPES (25 mM), gentamicin sulfate (50 μg/ml) and 0.5% Albumax II (Invitrogen). The cultures were synchronised by haemolysis of mature, late trophozoite-stage parasitised erythrocytes by suspension in 9 volumes of a 5% sorbitol solution at room temperature for 5 min. Cells surviving the treatment were used to set up new cultures and the process repeated where necessary to achieve a tighter synchronisation [16]. Given that synchrony is never perfect, time zero for the erythrocytic cycle was taken as the point where ca. 90% of infected erythrocytes were ring form, with the remainder as very late schizonts. To obtain free parasites, synchronised cultures, normally in the late (mature) trophozoite stages (ca. 30 h into the cycle), were quickly lysed with 0.05% (w/v) saponin at room temperature, permeabilising the red cell and parasitophorous membranes and leading to haemolysis [17,18]. Any remaining unlysed red cells were further treated with a PBS wash containing the same amount of saponin. The freed parasites were then resuspended in the appropriate buffer with or without glucose, depending upon the purpose of the assays. Saponin-freed parasites that are glucose-replete maintain intracellular levels of ATP for periods of at least 30 min and do not show evidence of leakage [19].

### 2.3 Uptake assay of radiolabelled folates

Folate uptake/export assays were performed on free parasites, parasitised red cells or uninfected red cells, as appropriate. Cell numbers were estimated with a haemocytometer. Normally preparations containing 10^7^–10^8^ parasites were used for each assay point (or ca. 10^7^–10^8^ uninfected red cells). Prior to uptake assays by naked parasites, infected cells were lysed in 0.05% saponin and the freed parasites washed either in PBS or folate/pABA depleted RPMI 1640, depending upon the experiment. Wash steps were also performed with different buffered or non-buffered isotonic salt solutions where necessary as indicated in the relevant text.

All cells, washed extensively to remove folate present in the culture medium, were mixed with or without glucose (20 mM final concentration) and inhibitors as appropriate, together with radiolabel (normally 1 μCi/ml, equivalent to 38 nM, unless otherwise stated) to make up a total volume of 100 μl with PBS or alternative buffer. All the components were prewarmed to 37 °C before the start of the assay, the reaction mix incubated at 37 °C normally for 30 min, and stopped by addition of 1 ml ice-cold PBS.

When assaying for folate uptake by whole parasitised red cells, these were spun down after the uptake period and the pellet washed with 1 ml ice-cold PBS at least three times to bring extracellular label down to the background level. The washed parasite pellet obtained by subsequent saponin treatment was then lysed with 0.02% SDS before counting. Uptake by naked parasites was determined in the same way. Unless otherwise indicated, the uptake values represent labelled folate that has been imported into synchronised trophozoite stage parasites. All assays were routinely performed in triplicate on different batches of parasites unless otherwise indicated, and the data expressed as the mean ± S.D.

### 2.4 Extraction and affinity purification of folate derivatives

For characterisation of labelled folates, parasite pellets were washed three times in 1 ml PBS to remove any extracellular label and resuspended in 1 ml extraction buffer (0.1 M Tris–HCl, 2% ascorbic acid, pH 7.5), heated in a boiling water bath for 10 min, centrifuged at 10,000 × *g* for 10 min and supernatants stored at −20 °C until affinity purification and HPLC analysis, performed as described [20]. Unlabelled folic acid, folinic acid and PteG2 to PteG5 of the polyglutamated forms of folic acid were added into the extracted folates as internal standards. An identical aliquot of sample was also spiked with ^3^H-folinic acid in a second run to confirm the identity of the radioactive peak of folinic acid that had been extracted from the parasites.

### 2.5 pH measurements

^3^H-folinic acid uptake as a function of pH was measured in a combination HEPES-MES buffer system containing 20 mM of each component, and the salt concentration made up to 154 mM with NaCl. By taking the temperature coefficients of the buffers into account, pH values were adjusted at room temperature such that the apparent pH value at the assay temperature (37 °C) was exactly the pH required. Glucose was added to 20 mM as required. The internal pH of isolated parasites was measured as described [21] using the pH-sensitive fluorescent indicator BCECF-AM. Loading was achieved by incubating isolated parasites suspended at a cell density of 0.9–2.1 × 10^8^ cells/ml in folate and pABA depleted RPMI 1640 without Albumax II, and containing 10 μM BCECF-AM, for 10 min at 37 °C. The cells were then washed by centrifugation and resuspended in culture medium and used immediately. An aliquot of cells was transferred to an appropriate buffer in a 2 ml cuvette in the temperature-controlled chamber of a spectrofluorometer, maintained at 37 °C. The sample was measured at 520 nm after successive excitations at 440 and 495 nm. The ratio of the fluorescence emissions (excitation at 495 nm/excitation at 440 nm) was used to calculate the internal pH of the cells. A standard curve was prepared with parasites resuspended in 130 mM KCl, 10 mM NaCl, 1 mM MgCl_2_ and 40 mM of MES-HEPES buffer from pH 5.5 to pH 8 and 30 μM nigericin [21].

## 3 Results

### 3.1 Uptake of folates over the asexual erythrocytic cycle

**Fig. 1:**
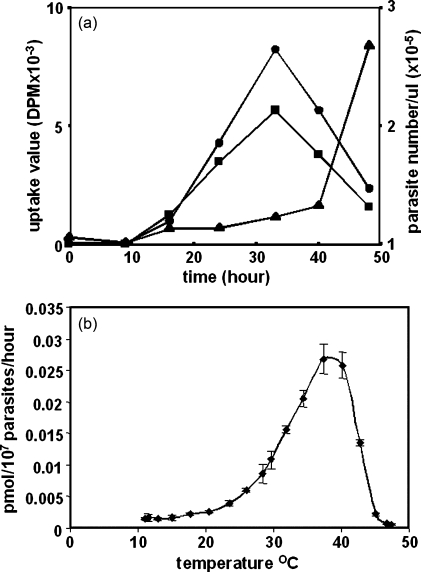
(a) Uptake of folates over the erythrocytic cycle of *P. falciparum*. Synchronised HB3 cultures (initial 4% parasitaemia) were sampled every 7–9 h, cells spun down, washed with pABA/folate depleted medium and incubated at 37 °C in 1 ml RPMI1640 containing 10 ng/ml each of pABA and tritium-labelled folinic acid (squares) or 5-MeTHF (circles) before extraction and counting of labelled folate from saponin-freed parasites (left vertical axis). The number of infected erythrocytes per unit volume present at each time point is also shown (triangles; right vertical axis). (b) Temperature dependence of folinic acid transport into *P. falciparum*. Saponin-freed parasites were washed with culture medium devoid of folate/pABA, then incubated in PBS, 20 mM glucose, 38 nM ^3^H-folinic acid. Parasites and reagents were prewarmed separately to the desired temperature in a thermal gradient block, mixed to initiate uptake and incubated for 1 h, before harvesting the parasites and counting imported label.

To monitor the ability of *P. falciparum* to salvage folate at different stages of the erythrocytic cycle, synchronised parasite cultures were incubated with different forms of radiolabelled folate. 5-Methyltetrahydrofolate (5-MeTHF) is the fully reduced form most abundant in human plasma [22] and thus the principal derivative available to the parasite in vivo. Folinic acid (5-formylTHF), although not known to be a normal physiological substrate for the parasite, is a more stable member of the fully reduced (active) forms that closely resembles 5-MeTHF and is used clinically (leucovorin). The capacity of ring stages to take up both of these compounds was very low but increased markedly ca. 15 h into the cycle as the parasites entered the trophozoite stages, peaking at around 30–35 h, before falling to very low levels again in mature schizonts (Fig. 1a). The numbers of parasites at the different stages were monitored throughout such time-courses and showed that uptake of folate increased as the parasites proceeded through the trophozoite stages against a near-constant number of infected cells, as expected for a synchronous culture. At around 40 h, some schizonts began to release merozoites, leading to a sharp rise in the total number of infected cells, but the uptake from this population continued to diminish, consistent with lower levels of uptake for both rings and schizonts, relative to trophozoites (Fig. 1a). To check that the marked reduction of uptake in the latter stages of the cell-cycle (beyond 40 h) was not due to parasites becoming unhealthy or inviable, the phenomenon was also monitored through a subsequent cycle, where the same pattern was repeated (data not shown). Only minor differences were seen between parasite clones of different lineages, and labelled folic acid, the stable oxidised form extensively used experimentally to study folate metabolism and a common dietary supplement, gave qualitatively similar results to the reduced folates. This pattern strongly suggested that folate uptake is a process regulated by *P. falciparum*, likely to involve a transport mechanism more complex than facilitated diffusion. On the basis of the data above, parasites at the late trophozoite stage of synchronous cultures were used to investigate the characteristics of uptake in subsequent experiments, using ^3^H-folinic acid as the label, as this is considerably more stable than 5-MeTHF, is more readily available in labelled form, and has been shown in earlier studies to provide a better source of cofactor than equimolar amounts of folic acid [23].

Uptake of folinic acid into the parasite was also markedly dependent upon temperature. Only a very low level of transport was observed between 10 and 25 °C, over which the parasites are viable, but metabolically retarded. The rate of uptake increased dramatically above ca. 30 °C, peaking quite sharply between 37 and 39 °C, but was abolished entirely above 45 °C, a temperature at which the parasites are no longer viable. Data for free parasites are shown in Fig. 1b; very similar profiles were obtained for infected erythrocytes with the same optimum temperature (data not shown). This demonstrated that active parasite metabolism in live organisms at their normal growth temperature is required for folate to be imported to a significant degree.

### 3.2 Kinetics of folate uptake

**Fig. 2:**
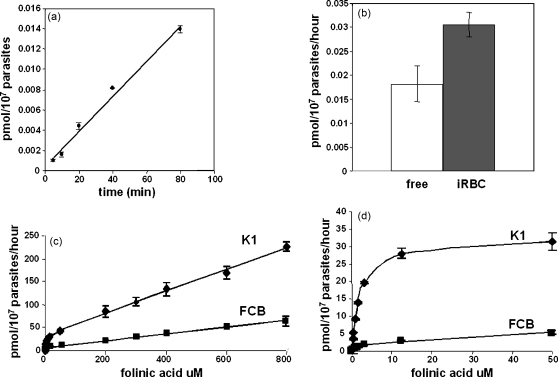
(a) Time dependence of folinic acid uptake illustrating linearity over 80 min. (b) Rate of uptake into naked parasites (free) and an equal number of parasites within erythrocytes (iRBC). (c) Concentration dependence of uptake illustrating the non-linear (saturable) and linear (non-saturable) components for K1 (diamonds) and FCB (squares), and (d) the saturable components for K1 and FCB compared. Two independent experiments each in triplicate for each of K1 and FCB yielded the kinetic parameters quoted in the text.

Assays of the time-dependence of folate uptake into trophozoites showed that it was linear (*R*^2^ ≥ 0.98) over a period of at least 80 min (Fig. 2a). In subsequent experiments, unless otherwise indicated, a standard incubation time of 30 min was therefore adopted to ensure that adequate counts for accurate measurements were obtained, whilst remaining well within the window of linearity. Uptake rates of folinic acid into parallel samples of free parasites and infected red cells in equal numbers were comparable, with somewhat higher levels measured in the latter (Fig. 2b), indicating that the rate-limiting step is not passage across the erythrocytic membrane, but rather across the parasite plasma membrane. In free parasite cells, the folate uptake process exhibited saturable and non-saturable components (Fig. 2c), indicative of more than one mechanism. In low concentrations of the substrate (in the region of 0 to 15 μM, which encompasses the normal range of human plasma folate, 30–40 nM [24]), folinic acid uptake is non-linear, consistent with the operation of a carrier-mediated process (Fig. 2c). At higher concentrations up to mM levels, uptake became linear, a phenomenon also observed in other folate transport systems [25,26], including that of *Toxoplasma* [6]. Subtraction of the linear component yielded *K*_m_ values for the saturable process of 3.4 ± 0.8 and 2.4 ± 0.3 μM, respectively, for FCB and K1 parasite strains (Fig. 2d). Interestingly, while their *K*_m_ values were closely similar, the $v_{\text{max}}$ observed for K1 (34.7 ± 2.6 pmol/10^7^ parasites/h) was ca. 10-fold higher than that for FCB (3.3 ± 0.3 pmol/10^7^ parasites/h).

### 3.3 Export of folinic acid

**Fig. 3:**
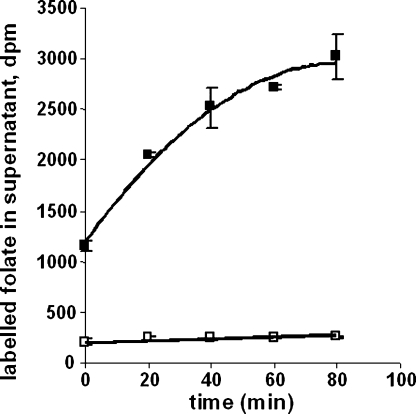
Export of folinic acid from uninfected erythrocytes (filled squares) and free parasites (open squares). ^3^H-folinic acid detected in the supernatant of the incubation mixture as a function of time after the initial 1 h uptake period into uninfected or infected erythrocytes and removal of extracellular label.

Export of folinic acid from free parasites was compared with that from uninfected cells. Uptake was first allowed to proceed into either infected or uninfected erythrocytes for 1 h in PBS plus 20 mM glucose at 37 °C with labelled folinic acid. After incubation and washing, cells were resuspended in the same buffer without label and incubated for different times (Fig. 3). Cells were then spun down and the radioactivity in the supernatant measured. To measure label exported from free parasites, they were first released from erythrocytes with 0.05% saponin and then washed and treated in a similar manner as for the uninfected red cells. The level of label in the supernatant of the red cell aliquots tripled over the 80 min incubation time, indicative of facile export. However, export of the label from free parasites was much less rapid, only increasing by ∼20% over the same period.

In the case of *Lactobacillus salivarius*, folate internalised over a similar period cannot be released from the cells and is recovered in cell extracts primarily in polyglutamated forms that play an important role in the retention and accumulation of intracellular folate [27,28], and previous studies using *P. falciparum* have demonstrated the presence of polyglutamated forms of folate produced from labelled monoglutamated precursors [2,23,20]. To examine whether the slow rate of export seen above was due to trapping by polyglutamation, the folinic acid internalised after 1 h was then extracted and analysed by HPLC on a C18 column [23,20]. The elution profiles indicated that the imported folinic acid had not been significantly modified by this stage, as no labelled polyglutamated folates could be detected in the cell extracts after this relatively short period of incubation, nor was there evidence of any conversion of label to another form of folate (data not shown). These results, together with our earlier data, where overnight incubations were required to detect polyglutamated forms [23,20], suggest that although these forms of folate probably play an important role in ultimately retaining the folates in malaria parasites and modulating the affinity of different enzymes for them, conversion to such forms is not rapid and appears not to play any immediate role in the salvage process. The low rate of net export from the parasite is however consistent with the operation of a transport system that actively imports folates into the parasite, where they are efficiently retained (see below).

### 3.4 The dependence of folate uptake on carbon source

**Fig. 4:**
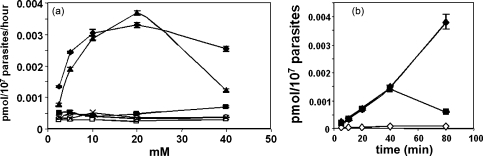
(a) Effect of varying concentrations of different sugars on the uptake of ^3^H-folinic acid into infected erythrocytes after incubation at 37 °C for 1 h. Filled diamonds, d-glucose; filled triangles, d-fructose; crosses, l-glucose; filled squares, 6-deoxy-d-glucose; open squares, d-galactose; open circles, d-xylose. (b) Effect of glucose on ^3^H-folinic acid uptake by infected erythrocytes as a function of time. Open diamonds, no glucose; filled squares, 2.5 mM glucose; filled diamonds, 20 mM glucose.

To investigate possible metabolic requirements for folate uptake, its dependence on glucose and other sugars was studied in parasitised red cells. In the absence of glucose or at concentrations <1 mM, only a very low level of import was measurable. As the glucose concentration was raised, a marked increase in imported folinic acid was observed, peaking around 20 mM (Fig. 4a). This corresponds closely to the level of glucose in standard *P. falciparum* culture medium (22 mM), empirically determined historically to give optimal levels of growth. Time course experiments also showed that the higher the glucose concentration, the longer the uptake process could be sustained by the parasites (Fig. 4b).

d-Glucose could be replaced by the alternative hexose sugar d-fructose, which can also be utilised in the glycolytic pathway of *P. falciparum* [29,30], but substitution by sugars that cannot, i.e. l-glucose, d-galactose, d-xylose or 6-deoxy-d-glucose, almost completely abolished uptake (Fig. 4a). Cellular energy in *P. falciparum* is predominantly provided by glycolysis, with the parasite using ca. 100 times more glucose than uninfected erythrocytes to supply their considerable energy needs [31], and uptake of folate is thus clearly dependent upon the operation of normal levels of intracellular energy metabolism.

### 3.5 Effect of pH on folate uptake

**Fig. 5:**
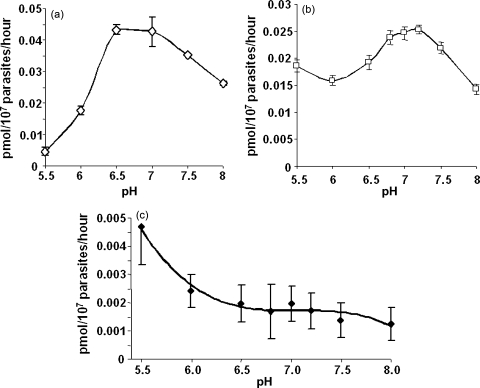
Effect of pH on the uptake of ^3^H-folinic acid into (a) infected erythrocytes in the presence of 20 mM glucose, (b) free parasites in the presence of 20 mM glucose and (c) free parasites in the absence of glucose, after repeated washes with 154 mM NaCl, to eliminate pH gradient changes across the parasite membrane caused by proton pumping.

In the presence of glucose or other sugar susceptible to glycolysis, as in the above experiments, the malaria parasite has a higher cytoplasmic pH (7.2–7.4) and a lower parasitophorous vacuole pH (6.9), produced by proton pumping activity on their plasma membrane [21,32]. Such pumping is necessary to prevent the build-up of intracellular acidity, and the higher proton concentration thus created outside the cell can be utilised for the purposes of nutrient transport [21]. This prompted us to investigate the effect of pH on the uptake of folinic acid. For this, a HEPES-MES buffer system was used that provided a wider range of buffering capacity than either buffer alone. The HEPES-MES system contained 20 mM of each component and the salt concentration was made up to 154 mM with NaCl. Initially, glucose (20 mM) was included in all wash steps and assay reactions. For parasitised red cells under these conditions, the optimal pH was 6.5–7.0, uptake then decreasing linearly with increasing pH between pH 7.0 and 8.0 (Fig. 5a), with a similar pattern being observed for free parasites over this range (Fig. 5b). However, the transmembrane pH gradient generated in this way is dynamic, subject to change by normal cellular metabolism. To avoid this potentially confounding effect, similar experiments were also conducted in the absence of glucose, thus cutting off the energy supply for the proton pump(s), with the desired buffering and osmotic requirements maintained by NaCl, HEPES and MES. A series of artificially created, constant transmembrane proton gradients were established in this way for the uptake assay. Saponin-released parasites were washed thrice with 154 mM NaCl to remove folate and other nutrients and to effect exhaustion of the glucose, thereby bringing all parasites in a given sample to the same internal pH. The results showed that under these conditions, the uptake of folinic acid is positively correlated with the transmembrane pH gradient over a wider range (Fig. 5c). The lower the pH of the resuspension buffer, the greater the pH gradient thereby provided, which in turn yielded a higher uptake of folinic acid. Above pH 6.5, the pH response curve flattened out and exhibited a relatively constant, low level of uptake. This inflection point may correlate with the internal pH of the parasites when depleted of glucose under the above conditions [21]. In some cases this was confirmed by measuring the internal pH value with BCECF-AM as described in Section 2. Attempts to directly measure a change in pH as a result of folate import in the absence of glucose and using a minimal buffer concentration were unsuccessful, possibly because of the very small quantities that are taken up and the relatively slow rates involved.

### 3.6 Effect of perturbing the plasma membrane proton gradient on folate uptake

**Fig. 6:**
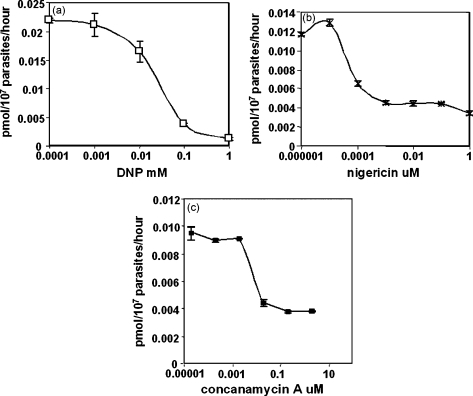
Inhibition of ^3^H-folinic acid uptake by disruption of the proton gradient using (a) 2,4-dinitrophenol; DNP, (b) nigericin and (c) concanamycin A. Parasites were released from red cells by saponin treatment and washed with HEPES-MES buffer, pH 7.2, with a total ionic strength of 154 mM + 20 mM glucose, to remove medium folate, nutrients and cell debris. The assays were performed in the same buffer at 37 °C for 30 min.

2,4-Dinitrophenol (DNP) is a widely used ionophore in the uncoupling of oxidative phosphorylation from electron transport on the mitochondrial membrane, as well as in studies of transport across the plasma membrane. Mechanistically, it provides an efficient by-pass route for protons that dissipates their gradient and hence the proton motive force. Here it was found that it also inhibits folinic acid uptake into parasite cells, with an IC_50_ of ca. 26 μM (Fig. 6a and Table 1), again suggesting that this uptake is intimately coupled with the energy metabolism of the parasites. Similarly, the K^+^/H^+^ antiporter nigericin had a profound effect on the uptake of label, which decreased in a dose-dependent manner, with a marked drop in the 100 pM range. Interestingly, this effect levelled off at around 1 nM to about 75% inhibition and showed little further drop up to 1 μM nigericin (Fig. 6b), suggesting that the major, but possibly not sole, factor determining the uptake of folinic acid is the magnitude of the pH gradient. A similar phenomenon was observed when the V-type ATPase inhibitor concanamycin A [33] was employed. Such an enzyme has been identified as the principal source of proton pumping in *P. falciparum* [21,32]. This inhibitor was effective at sub-micromolar concentrations (IC_50_ ca. 16 nM; Fig. 6c), but only abolished ca. 60% of folate uptake, with no further effect beyond about 100 nM.

### 3.7 Metal ion dependency of folate uptake

To investigate whether folate uptake was affected by other cations, in addition to its dependence on the proton gradient demonstrated above, parasitised cells were first washed with 100 volumes of buffer containing either (i) PBS (the buffer in which other uptake experiments were conducted), (ii) a near-physiological concentration of Na^+^ (130 mM NaCl, 20 mM Tris–HCl, pH 7.0), (iii) an equivalent concentration of K^+^ (130 mM KCl, 20 mM Tris–HCl), (iv) the non-ionic polyol mannitol (130 mM mannitol, 20 mM Tris–HCl), or (v) Tris alone (150 mM Tris–HCl). Washes were repeated thrice to reduce the contribution of pre-existing salts to an insignificant level compared to those in the assay buffer. Uptake rates of folinic acid from all five of these solutions were similar (<±20% from the mean). Also, the presence of EDTA up to 5 mM or Mg^2+^ up to 10 mM had only a minor effect (data not shown). It thus appears unlikely that sodium, potassium or divalent metal ions are critically involved in the co-transport of folate into the malaria parasite.

### 3.8 Effect of antifolate drugs and other inhibitors on folate uptake

A range of other potential inhibitors and competitors were tested for their ability to affect transport of folate into the parasite (Table 1). Antifolate drugs exert their effect principally by binding to target enzymes (DHFR and DHPS) in the folate pathway, lowering pools of reduced folate and thus compromising key metabolic steps, especially DNA synthesis. However, in principle, they might also exhibit an inhibitory capability by interfering with the uptake of exogenous folate [4]. To investigate this possibility, uptake of labelled folinic acid into free parasites was monitored in the presence of the DHFR inhibitors pyrimethamine (PYR) and methotrexate (MTX), and the DHPS inhibitor sulfadoxine (SDX). Over a range of 0.4 nM to 40 μM for PYR and 0.3 nM to 322 μM for SDX, no inhibitory effect on folate uptake could be detected (the highest concentrations are ca. 900 and 500 times greater than the respective IC_50_ values for these drugs measured for growth inhibition). However, MTX did show inhibition of folinic acid uptake, consistent with its much closer structural relationship to folate than either Pyr or SDX. This was seen clearly when MTX (IC_50_ of ca. 13 μM) was compared to unlabelled folinic acid (2.6 μM), 5-MeTHF (3.5 μM) and folic acid (19.8 μM), where the drug competed with uptake of the label to a similar degree. The specificity of the transport process across the parasite plasma membrane suggested by the above results is consistent with a complete lack of competition with pantothenate (Table 1), the precursor of coenzyme A and an essential parasite nutrient [21], which is also a small but unrelated organic acid with a molecular weight about half that of folate.

**Fig. 7:**
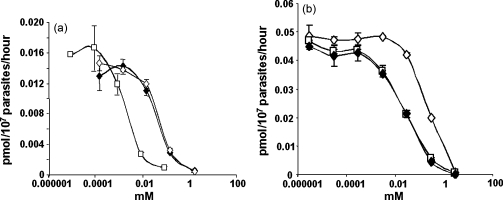
Inhibition of ^3^H-folinic acid uptake by channel blockers. (a) Free parasites: open diamonds, probenecid; filled diamonds, furosemide; open squares, 5-MeTHF (control). (b) Infected erythrocytes: open diamonds, probenecid; filled diamonds, furosemide; open squares, probenecid + furosemide at equal concentration.

To further explore the nature of the transporters involved in folate uptake, we employed two known channel blockers, probenecid and furosemide. Probenecid is known to inhibit folate uptake via an anion carrier in mammalian cells at mM levels [34,35], and is able to sensitise parasites to antifolate drugs [15]. Furosemide has been extensively used in nutrient transport studies of the malaria parasite, as it efficiently inhibits the so-called new permeability pathways (NPP) [36–38], which first appear in the erythrocyte membrane of parasitised red cells about 12–15 h into their intraerythrocytic development. The NPP show characteristics of anion-selective channels, but are of broad specificity and increase erythrocyte permeability to a range of small molecular weight anions, cations and other species [39–42], including essential nutrients such as pantothenate [11]. In experiments conducted on free parasites, where the NPP are not relevant, probenecid strongly inhibited folinic acid uptake with an IC_50_ of ca. 46 μM, and interestingly, furosemide was just as effective (IC_50_ of ca. 39 μM) (Fig. 7a and Table 1). When parasitised erythrocytes were used in the assay, furosemide blocked uptake effectively with an IC_50_ value of ca. 21 μM, similar to that for the free parasite, whereas probenecid was now less effective (IC_50_ of ca. 187 μM). The two drugs were assayed individually and also in combination under identical conditions, but no synergy was apparent between them (Fig. 7b). Indeed, the apparent absence even of an additive effect showed that when both drugs are present in equimolar amounts, the overall effect is equivalent to that seen with the drug of higher potency, i.e. furosemide. This suggests that the two drugs are likely to be acting on the same target. Given this and the above results with free parasites, where the target involved is shown by our prior experiments to behave as a specific folate transporter, it seems probable that the major effect of these two drugs on infected erythrocytes is also at folate transporters on the erythrocyte membrane, rather than the NPP, although we cannot exclude transport via the latter acting to supplement the endogenous capacity of the erythrocyte to import the higher levels of folate required by the parasite.

## 4 Discussion

Although *P. falciparum* possesses the biosynthetic machinery required to produce folate derivatives de novo, folate salvage is also an important aspect of the parasite's metabolism [7]. We present several lines of evidence to demonstrate that transport of exogenous folate into *P. falciparum* is primarily a carrier-mediated process dependent upon cellular energy and the trans-plasma membrane proton gradient. Uptake over the erythrocytic cycle is clearly regulated, with the highest capacity occurring at the late trophozoite stage. This coincides with a period of intense metabolic activity and the onset of multiple rounds of DNA replication, for which folate cofactors are essential, and resembles the pattern seen in the folate auxotroph *Leishmania*, a parasite that also imports folates maximally in the logarithmic growth phase [43–45]. The exogenous folate requirement of *P. falciparum* can be satisfied in vitro by folic acid, 5-formylTHF (folinic acid) and 5-MeTHF, similar to that of the folate-requiring bacterium *Lactobacillus casei*, but not *L. salivarius*, which cannot use the last of these [27]. The process also exhibits a marked temperature dependence, characteristic of a membrane carrier system, with significant uptake only occurring close to the parasite's optimum growth temperature of 37 °C. Once imported, net loss in the reverse direction appears to be a slow process, but over the short periods monitored here, did not involve a detectable level of polyglutamation that would trap the folate within the cell, although a significant portion of imported folate is ultimately modified in this way [2,23,20]. This may imply that the role of polyglutamation in this case may be more related to enzyme-substrate recognition, rather than intracellular trapping.

The *K*_m_ values of ca. 2–3 μM that we measure for the saturable binding component for folinic acid of two strains of *P. falciparum* are higher than those of 0.4 and 42 nM, respectively, for folic acid uptake into *Lactobacillus* [27], *Xenopus* oocytes [46], and of 250–700 nM for such uptake into *Leishmania* parasites, depending upon the species [47]. This may reflect the absolute dependence of these three organisms on folate salvage, whereas *P. falciparum* also possesses an active folate biosynthesis pathway. The $v_{\text{max}}$ values we observed are of the same order as the rate of uptake of the vitamin pantothenic acid into the parasite [11] but considerably slower than that of glucose [48,49,10], presumably reflecting the great difference between reusable cofactors required in limited quantities and the enormous consumption of the latter as the principal source of energy. The more rapid uptake of folate into the antifolate-resistant strain of K1 compared to the sensitive FCB strain, as indicated by their quite different $v_{\text{max}}$ values, could result from the selection of parasites better able to cope with drug-mediated inhibition of folate metabolism by increasing their salvage efficiency, in addition to the familiar mutation events in genes encoding folate pathway enzymes that reduce drug binding to the latter [50]. Moreover, if the mutant enzymes (DHFR-TS and PPPK-DHPS) from such parasites process their substrates in vivo with a lower efficiency than in the wild-type, compensation by an increased flux through the salvage pathway might be important for healthy growth, even in the absence of drug. These observations merit extension to other parasite strains with differing responses to antifolate drugs, to further test this hypothesis.

In principle, import of folates into the infected erythrocyte could occur via the new permeation pathway(s) (NPP) induced in the host cell membrane by the growing parasite [39,41,42]. However, mammalian cells also exhibit two types of endogenous folate transport system, the reduced folate carriers (RFC), which possess multiple transmembrane domains and have a greater affinity for the reduced folates than for folic acid, and the folate receptors (FR), which are GPI-anchored external folate binding proteins that mediate transport of both folic acid and 5-MeTHF with high specificity [51–53]. In mature erythrocytes, the FRs lose functionality with age [51] and the RFC is thought then to be the major transporter type [25,53]. Whatever the exact nature of the relevant transporters on the erythrocyte membrane, they appear not to represent a bottleneck for folate uptake into the parasite, as indicated by our rate experiments. Nor would the parasitophorous vacuolar membrane, which is highly porous and freely permeable to small molecules [54]. The rate-limiting step is therefore passage through the parasite plasma membrane, a process that is strongly inhibited in free parasites both by probenecid, a proven inhibitor of folate transport in mammalian cells [34] and furosemide. Given the similarity in these anion channel blockers (both are benzoic acid derivatives of comparable size with aminosulfonyl substituents on the benzene ring) and the evidence that they target the same sites on infected erythrocytes, we conclude that their major effect with respect to folate uptake at the erythrocyte membrane is likely to be at endogenous folate transporters rather than the NPP. Consistent with the view that the transporters involved in the parasite plasma membrane also have a high degree of specificity for folate, we note that the antifolate drugs PYR and SDX, which have some structural features in common with folate, do not compete with uptake of folinic acid even at very high concentrations. In contrast, the anticancer antifolate MTX, which is a much closer structural analogue to folic acid than either PYR or SDX, does inhibit uptake to a similar extent to the unlabelled folates themselves (μM range) in competition experiments. We note that the lack of inhibition of folate uptake by PYR that we report here rules out our earlier hypothesis [4] that such inhibition might contribute to the synergy observed between PYR and SDX.

We have demonstrated that folate uptake into *P. falciparum* is dependent upon the presence of d-glucose or other sugar that can be metabolised via glycolysis, a process that is essential to maintain the proton and electrochemical gradients across the parasite plasma membrane [21,8]. d-Fructose can be phosphorylated by hexokinase to fructose 6-phosphate, the substrate of the third glycolytic enzyme, phosphofructokinase-1. Interestingly, although fructose is only about half as efficient as glucose in maintaining overall growth of the parasite [30], it was as efficient as glucose in supporting folate import on a molar basis in our experiments. This may reflect the fact that only a small fraction of the glycolytic flux is likely to be necessary to support import of the relatively low amounts of exogenous folate required by the parasite. Of the other sugars we tested, which were all unable to support uptake, l-glucose is stereochemically excluded, while 6-deoxy-d-glucose and d-xylose can bind to hexokinase but cannot be phosphorylated. d-Galactose, derived principally from lactose hydrolysis, can be converted to glucose 6-phosphate in mammals via a four-step mechanism involving uridine diphosphate derivatives, but its inability to support folate import here would be consistent with an apparent lack of galactokinase and the other enzymes required for this conversion in the predicted proteome of *P. falciparum*.

The dramatic reduction of folate transport in the absence of a metabolisable sugar mirrors the rapid decline of intracellular ATP concentration and drop in the internal pH of the parasite seen in the same circumstances [21]. This suggested that inward movement of folates depends upon maintenance of a pH (interior alkaline) or electrochemical gradient (interior negative), or both, across the plasma membrane. Such gradients can be generated by membrane-bound ATPases, and a range of organic anions move into bacterial cells using proton symport. To differentiate between movement of folate via symport with protons or another cationic species, we examined the influence of the latter, but saw only minor changes in uptake rates in the presence or absence of Na^+^, K^+^, Mg^2+^ or EDTA. This differs from the folate transport system in *Lactobacillus*, which although similarly dependent upon glucose, also has a requirement for divalent cations [55,27].

The pH optimum that we observe for folate import (6.5–7.0) corresponds well with that (pH 6.9) measured just outside of the parasite in infected erythrocytes [32], 0.4 units lower than the pH of 7.3 in the parasite cytoplasm [21,32]. The primary mechanism for exporting protons in *P. falciparum* is thought to be via a V-type H^+^ ATPase on the plasma membrane [21,32], and consistent with this, we found that concanamycin A, a specific inhibitor of such ATPases [56], inhibited folate uptake in the low nM range. Genes encoding the A and B subunits of a V-ATPase have been identified in *P. falciparum* [57,58] and immunological studies indicate that this enzyme is expressed throughout the erythrocytic cycle [32]. Interestingly though, a significant acidification of the extracellular space just outside of the parasitophorous vacuole could only be measured in trophozoites [32], the stage of the life-cycle at which we observe the highest levels of exogenous folate uptake. The substantial proton gradient that is generated via the V-ATPase is the major contributor to an inwardly negative membrane potential of ca. −95 mV [8], but protons are also extruded in a symport system together with the large amounts of lactate generated by parasite glycolysis [12]. This may explain why we did not observe 100% inhibition of uptake with concanamycin, even at μM concentrations, as it blocks only the major portion of the total proton export.

The importance of the proton gradient across the plasma membrane was emphasised by the sensitivity of folate uptake to the ionophores DNP and nigericin, both of which strongly perturb this gradient. In particular, we find that folate uptake is exquisitely sensitive to nigericin, with substantial inhibition at sub-nM levels. Whereas DNP collapses the pH gradient completely by enabling free passage of protons in both directions, the K^+^/H^+^ antiporter mechanism of nigericin is presumably limited by the degree to which the K^+^ gradient can be perturbed, which may explain why we also did not observe 100% inhibition of uptake with this compound. Although the profiles observed for nigericin and concanamycin A can be explained in principle by assuming that neither abolishes the pH gradient completely, we cannot exclude the possibility that there is also a component of folate transport independent of this gradient. This scenario is suggested by the fact that a low level of transport persists even when the external pH is set at the same, or a higher, level than the internal pH of parasites deprived of glucose, and would also be consistent with the two-phase nature of the concentration dependence of uptake. At first sight, this picture is difficult to reconcile with the data for DNP, which by insertion into the membrane, permits rapid equilibration of proton concentrations on each side, but we note that complete abolition of uptake only occurs at ca. 1 mM, a concentration at which quite profound changes to the membrane might be expected, possibly impacting on any proton gradient-independent component.

A plausible model encompassing our observations would be that folate uptake at the parasite plasma membrane is mediated by a transport complex or multi-binding site protein possessing a binding domain specific for folate recognition and a probenecid/furosemide-sensitive proton symport channel through which the folate is internalised. To date, no folate transporters have been functionally identified in *P. falciparum*, but bioinformatics studies [59] predict the existence of 3 candidate proteins (gene loci MAL8P1.13, PF11_0172 and PF10_0215 in PlasmoDB; http://www.plasmodb.org) that are related to biochemically verified molecules in *Leishmania* [47,45], *Synechocystis* and *Arabidopsis* [60]. Related putative candidates have also been identified in *Toxoplasma gondii* [6]. This sub-family of folate-biopterin transporters (FBT) are thought on bioinformatic grounds to function as proton symporters [59], and on this basis, the data we present here would be consistent with one or more of these proteins providing the machinery for folate uptake in *P. falciparum*, which might ultimately present a novel target type for parasite inhibition. We are currently investigating the genes encoding these candidate molecules in knockout studies to better understand the relationship between the phenomena reported here and the parasite proteins involved.

## Figures and Tables

**Table 1:** Effect on uptake of labelled folinic acid into free parasites by a range of competitors/inhibitors under standard assay conditions

Compound	IC_50_ ± S.D. (μM)	*n*
Folinic acid	2.6 ± 0.1	3
5Me-THF	3.5 ± 0.3	3
Folic acid	19.8 ± 1.1	3
MTX	13.4 ± 1.0	3
Pyr	No inhibition	
SDX	No inhibition	
Pantothenic acid	No inhibition	
DNP	26.5 ± 2.1	3
Nigericin	0.12 ± 0.004 (nM)	4
Concanamycin A	15.6 ± 0.6 (nM)	6
Furosemide	38.9 ± 2.9	3
Probenecid	45.7 ± 3.3	3
